# Programming of Fetal Insulin Resistance in Pregnancies with Maternal Obesity by ER Stress and Inflammation

**DOI:** 10.1155/2014/917672

**Published:** 2014-06-30

**Authors:** Francisco Westermeier, Pablo J. Sáez, Roberto Villalobos-Labra, Luis Sobrevia, Marcelo Farías-Jofré

**Affiliations:** ^1^Cellular and Molecular Physiology Laboratory (CMPL), Division of Obstetrics and Gynecology, School of Medicine, Faculty of Medicine, Pontificia Universidad Católica de Chile, P.O. Box 114-D, 8330024 Santiago, Chile; ^2^Facultad de Ciencia, Universidad San Sebastián, 7510157 Santiago, Chile; ^3^Advanced Center for Chronic Diseases (ACCDiS), Faculty of Chemical & Pharmaceutical Sciences & Faculty of Medicine, University of Chile, 8380492 Santiago, Chile; ^4^Facultad de Ciencias de la Salud, Universidad San Sebastián, 7510157 Santiago, Chile; ^5^University of Queensland Centre for Clinical Research (UQCCR), Faculty of Medicine and Biomedical Sciences, University of Queensland, Herston, 4006 QLD, Australia

## Abstract

The global epidemics of obesity during pregnancy and excessive gestational weight gain (GWG) are major public health problems worldwide. Obesity and excessive GWG are related to several maternal and fetal complications, including diabetes (pregestational and gestational diabetes) and intrauterine programming of insulin resistance (IR). Maternal obesity (MO) and neonatal IR are associated with long-term development of obesity, diabetes mellitus, and increased global cardiovascular risk in the offspring. Multiple mechanisms of insulin signaling pathway impairment have been described in obese individuals, involving complex interactions of chronically elevated inflammatory mediators, adipokines, and the critical role of the endoplasmic reticulum (ER) stress-dependent unfolded protein response (UPR). However, the underlying cellular processes linking MO and IR in the offspring have not been fully elucidated. Here, we summarize the state-of-the-art evidence supporting the possibility that adverse metabolic postnatal outcomes such as IR in the offspring of pregnancies with MO and/or excessive GWG may be related to intrauterine activation of ER stress response.

## 1. Introduction

The global epidemic of overweight and obesity is defined by the World Health Organization (WHO) as abnormal or excessive body fat accumulation that presents a risk to health. WHO defines normal weight, overweight, and obesity as a body mass index (BMI, calculated as ratio of weight in kg/height in m^2^) of 18.5–24.9, 25–29.9, and 30 or greater, respectively. Obesity is further categorized by BMI into class I (30–34.9), class II (35–39.9), and class III or extreme obesity (≥40) [1]. BMI data from the WHO show that 43% of countries with recent nutritional information reported that half or more of their adult population has a BMI ≥ 25 [2]. The increasing prevalence of this nutritional problem is associated with many diet-related chronic diseases, including diabetes mellitus, cardiovascular disease, stroke, hypertension, and certain cancers. In pregnancy, obesity is associated with various perinatal morbidities [3], including diabetes (pregestational and gestational), cesarean delivery, gestational hypertension and preeclampsia, congenital anomalies, macrosomia (birthweight > 4000 g), and maternal or fetal mortality [4, 5].

In addition to the perinatal complications of obesity during pregnancy, increasing epidemiological evidence suggests persistent and deleterious effects of maternal obesity (MO) on the offspring and through intrauterine programming [6, 7]. However, the underlying mechanisms that could explain a potential link between MO and risk of problems such as insulin resistance (IR) in the offspring remain unclear. In overweight and obese individuals, nutrient excess is associated with a chronic inflammatory [8, 9] and cellular stress [10] signaling network involved in the adaptive response to persistent overload of glucose, amino acids, and free fatty acids (FFA) [11]. Adipose tissue produces circulating bioactive substances named adipokines (such as leptin, adiponectin, and resistin) and inflammatory markers (such as interleukin (IL) 6 and tumor necrosis factor *α* (TNF-*α*) [12]). These molecules are also implicated in the etiology of obesity-induced IR, based on common activation of stress-responsive proteins including c-Jun-NH2-terminal kinase (JNK), the inhibitor of nuclear factor kappa B (IKK), protein kinases C (PKC), and R (PKR) [12–14]. Growing evidence indicates that the cellular stress linking obesity and increased circulating and subcellular markers of IR implies a crucial role of the endoplasmic reticulum (ER) stress response [15–22]. The present review summarizes the findings supporting the hypothesis that adverse metabolic postnatal outcomes such as IR in the offspring of pregnancies with obesity and/or excessive gestational weight gain (GWG) are related to intrauterine programming and activation of the ER stress response.

## 2. Postnatal Effect of Maternal Obesity on the Offspring

Obesity and excessive GWG are recognized as independent risk factors for maternal and fetal complications [4, 5, 23]. Since the first publication by the Institute of Medicine in 1990 of GWG recommendations [24], there has been a 70% increase in the prevalence of prepregnancy obesity in the USA [4]. A large percentage of obese individuals will experience comorbidities during their life span, including the fertile age. Among the major general medical comorbidities are hypertension, cardiovascular disease, diabetes mellitus, hyperlipidemia, metabolic syndrome (a clinical condition associated with central obesity, hypertension, dyslipidemia, and IR), thromboembolic events, and cancer. In pregnancy, obesity is associated with various perinatal morbidities, including diabetes (pregestational and gestational), cesarean delivery, gestational hypertension and preeclampsia, congenital anomalies, macrosomia (birthweight > 4000 g), and maternal or fetal mortality [4, 5]. Prepregnancy obesity and excessive GWG have been implicated in an intergenerational “vicious cycle” of obesity [25]. Overweight or obese pregnant women have an increased probability of delivering macrosomic daughters, who are more likely to become obese themselves and deliver large neonates [26, 27]. In fact, GWG and birthweight are directly associated with BMI and risk of obesity in adolescence [28–30]. Based on these results, Oken et al. proposed that GWG guidelines should account for these influences of maternal nutrition on child weight [31]. The reported relationship was independent of other parental characteristics, potentially mediating peripartum factors, and abnormal dietary behaviors in the child, suggesting a role for the intrauterine environment in long-term offspring weight regulation. Interestingly, the association between maternal GWG and increased risk of adiposity in the offspring has been shown to emerge as early as 3 [31] or 7 years of age [32].

Considering the high prevalence of obesity in pregnancy and its association with gestational diabetes, there is an increasing interest in exploring the potentially negative influence of maternal overnutrition and elevated birthweight on the risk of disease in childhood and adulthood [25, 26, 41, 42]. Thus, it has been reported that children of obese women are more likely to develop IR later in life [43, 44]. Fraser et al. showed a detailed association of GWG and prepregnancy weight with offspring cardiovascular risk factors at the age of 9, in a large cohort of mother-offspring pairs from the Avon Longitudinal Study of Parents and Children (ALSPAC) [33]. In these studies, women who gained excessive weight during gestation were more likely to have offspring with greater BMI, waist size, fat mass, leptin, systolic blood pressure, C-reactive protein, and IL-6 levels and lower high-density lipoprotein cholesterol and apolipoprotein A levels (Table 1). Additional analysis demonstrated that excessive prepregnancy weight was also independently associated with greater offspring adiposity and adverse cardiovascular risk factors (Table 1) [34–38, 45, 46]. Epidemiological studies revealed that MO increases the incidence of metabolic syndrome in children [41, 47]. Moreover, the effect of MO on the susceptibility to obesity in offspring seems to be independent of gestational diabetes, as obese women with normal blood glucose have neonates with increased adiposity [39]. Interestingly, the same group has shown that MO is related to metabolic compromise already apparent at birth, characterized by reduced insulin sensitivity and increased serum inflammatory markers [26, 40]. Hence, maternal prepregnancy obesity and excessive GWG are independently related to an increased risk of obesity, IR, and very early markers of cardiovascular disease in the offspring. This evidence shifted our attention towards the gestational period as an extremely important intervention target in prevention of the obesity epidemic and its associated consequences such as IR and cardiovascular risk.

Ample evidence has indicated differential contributions of genetic and environmental factors in the development of noncommunicable diseases, such as obesity, diabetes mellitus, or cardiovascular diseases. In the case of obesity, the demographic shift of populations towards a fatty phenotype over a relatively short period of one or two generations argues against a major genetic contribution in favor of environmental or epigenetic mechanisms. In line with the concept of greater relevance of environmental factors, recent reports suggest that the prevention of childhood and adult obesity may need to begin even before conception [48–51]. Since pregnancy is a critical period of life, ethical considerations limit our ability to perform detailed mechanistic studies in humans. Therefore, animal models have been developed to address multiple questions in reproductive medicine.

Several animal models are used to study the mechanisms linking the altered prenatal environment in MO with the increased risk of obesity and other metabolic consequences in the developing offspring [52, 53]. Feeding animals a high fat diet (HFD) is a common model of overnutrition in pregnancy. Pups from rats on HFD during pregnancy and lactation, for instance, were shown to be heavier, fatter, and more hyperglycemic and moreover had higher hepatic lipid content at weaning than pups from rats fed a control diet [54]. In a similar mouse model, chronic maternal overnutrition was associated with hyperphagic behavior, reduced locomotion, increased adiposity, nonalcoholic fatty liver disease, and IR in the offspring at 3 and 6 months of age [55, 56].

Rodent models genetically predisposed to obesity are also used to evaluate the effects of MO. Genetically normal offspring of obese Agouti mouse dams, for example, were heavier than offspring from controls [57]. Interestingly, although adult weight did not differ between groups, female offspring of obese Agouti mothers had reduced *β*-pancreatic cell function and altered glycemic homeostasis [57]. Another transgenic model of maternal obesity is the heterozygous leptin receptor-deficient mouse (Lepr (db/^+^)) [58]. The pregnant Lepr (db/^+^) female is characterized by overeating, increased weight gain during pregnancy, and spontaneous development of gestational diabetes. Moreover, wild-type offspring show increased fetal growth and postnatal markers of hepatic insulin resistance, suggesting the occurrence of fetal programming [58]. Several research groups have been working to understand the mechanisms by which intrauterine metabolic alterations lead to particular phenotypes and influence susceptibility to obesity and metabolic diseases. The molecular mediators and signaling pathways that could be related to offspring metabolic phenotype (such as obesity and IR) are not fully elucidated. However, multiple inflammatory cytokines, hormones such as leptin or adiponectin, and nutrients such as glucose, free fatty acids (FFA), and triglycerides could be implicated in a mechanistic explanation of the increased metabolic risk in the offspring of MO [6, 14].

During normal intrauterine life, maternal insulin [59] and human insulin analog lispro (Humalog) [60, 61] are unable to cross the placenta, whereas maternal glucose is actively transferred to the fetus [62]. The developing human fetal pancreas responds to a glucose load by producing insulin, which acts as a fetal growth hormone in addition to its hypoglycemic effects. This is the basic concept of the “Pedersen's hyperglycemia-hyperinsulinism hypothesis” [63] to explain why offspring of diabetic mothers exhibit relatively higher birthweight [64, 65]. Maternal overnutrition produces maternal hyperglycemia, which increases fetal insulin secretion in a way similar to that observed in gestational diabetes [64–66]. Therefore, secondary fetal hyperinsulinemia is believed to be involved in the intrauterine programming of obesity and diabetes [41]. Prospective studies indicate that, at 6 years of age, as at birth, the greatest increase in weight-to-height ratio (relative obesity) was seen in children who experienced the greatest exposures to insulin in the uterus (as judged by amniotic fluid insulin concentration) [59]. Furthermore, animal studies show that systematic insulin administration to rats during pregnancy produces increased fetal growth [67], hyperinsulinemia, and impaired glucose tolerance [68].

Leptin may be implicated in the metabolic impairment observed in the offspring of MO and diabetes, as elevated circulating levels of this hormone are found in maternal and neonatal serum in association with these conditions [69]. However, in spite of the fact that placental transfer of leptin has been demonstrated* in vivo* [70], it is believed that umbilical levels of this circulating peptide are a marker of neonatal adiposity more than a modulator of fetal growth [69]. Several inflammatory cytokines are elevated in obese pregnant women [71] and have been postulated to be potential mediators of metabolic programming.

Consequently, the literature strongly suggests that altered metabolic phenotypes such as obesity and IR observed in the offspring of obese mothers could be partially explained by multiple mediators. It is likely that a model encompassing the multifactorial contributions of nutrient (such as glucose, fatty acid, and amino acid) and hormone (such as insulin and leptin) signals between the obese mother and the developing fetus would best describe the true mechanisms involved. The general question addressed in this review is how these factors induced by maternal obesity could modify insulin-dependent metabolic homeostasis in the offspring.

## 3. Insulin Resistance Mechanisms

Insulin is a key endocrine hormone that controls whole-body glucose, lipid, and protein homeostasis [72]. It also controls several other important processes such as cell growth, cell proliferation, survival, and differentiation [73]. Insulin mediates its biological effects via activation of insulin receptors A (IR-A) and B (IR-B) [74, 75] in major insulin target tissues [76], including human umbilical vein endothelial cells (HUVEC) [64] and human placenta microvascular endothelium (hPMEC) [65]. Subsequently, binding of insulin to IR-A and/or IR-B promotes its autophosphorylation and activation of the insulin receptor substrate family 1–4 (IRS1-4) [77, 78]. Phosphorylated IRS-1 (P-IRS-1) can bind adaptor proteins by linking its Src Homology 2 domain (SH2), such as p85 (regulatory subunit of phosphatidylinositol-3 or PI_3_K) and growth factor receptor-bound protein 2 (Grb-2). Thus, when the SH2 domain of p85 binds to IRS-1-P, the catalytic subunit p110 of PI_3_K is activated. In the same way, binding of SH2 domain of Grb-2 to P-IRS-1 activates the associated factor Son of sevenless (SOS). Next, activation of PI_3_K generates lipid mediators such as inositol triphosphate (IP_3_), which in turn initiates a cascade of signaling events dependent on protein kinases. These protein effectors include the IP_3_-dependent kinase 1 (PDK-1) and protein kinase B (Akt) [79]. Depending on the cell type, the insulin signaling pathway culminates in a series of different effects, such as glucose transporter that mediates the uptake in mediated uptake in liver cells [79] or activation of nitric oxide (NO) production in HUVEC [64] and hPMEC [65] in normal pregnancies. In a similar phosphorylation pathway cascade, activation of Grb-2/SOS involves activation of GTP-binding proteins Ras/Raf and mitogen-activated protein kinases (MAPK) [77]. Because both MO [27] and gestational diabetes [80] have been associated with decreased insulin sensitivity and increased IR status in the offspring, we will analyze how those insulin signaling mechanisms could be impaired in pregnancy.

IR is defined as a pathophysiological condition of underresponsiveness to normal insulin concentrations in target tissues such as adipose, muscle, liver, or cardiovascular tissues. Impaired insulin action is caused by reduced expression and/or function of its complex cellular response machinery [78]. Postreceptor defects in the intracellular insulin signaling pathway at different levels (such as in the mitochondria) may explain IR [81]. Whereas the insulin pathway branch dependent on PI_3_K has been thought of as being responsible for the metabolic and vasodilator effects in response to insulin stimulation, the Grb-2/SOS branch has been associated with the mitogen and vasoconstrictor actions of insulin [73, 78, 82]. Hence, abnormal predominance of the insulin derived from Grb-2/SOS signaling branch over the PI_3_K pathway has been associated with altered insulin effects on multiple tissues (such as liver, muscle, fat, and blood vessels). Interestingly, stress-activated protein kinases that phosphorylate IR or IRS-1 in serine or threonine residues are associated with inhibition of PI_3_K signaling and promotion of IR at metabolic and vascular levels [78].

In overweight and obese individuals, nutrient excess is associated with a chronic inflammatory and cellular stress signaling network involved in the adaptive response to persistent overload of glucose, amino acids, and FFA [11, 83, 84]. Circulating levels of adipokines (such as leptin, adiponectin, and resistin) and inflammatory mediators (such as IL-6 and TNF-*α*) are directly related to total body fat [12, 85]. These adipokines in turn are associated with autocrine and paracrine cell signaling alterations in response to obesity. All of these circulating products are implicated in the etiology of IR mediated by activation of stress-responsive proteins such us JNK, IKK, PKR, and PKC [12, 83, 85]. In fact, TNF*α* and FFA are potent activators of JNK, and increased concentrations of these mediators could explain the elevated function of this stress cascade in HFD and genetically obese mouse models [86]. Research which focused on the metabolic consequences of cellular stress in the context of IR development in obese individuals involves a crucial role for the ER stress response [15–20].

## 4. Endoplasmic Reticulum (ER) Stress Response

The ER is a complex intracellular membranous network that is essential for the synthesis and processing of secretory and membrane proteins [87]. It is highly sensitive to alterations in cellular environmental changes. It works as a quality control station that allows for transit of correctly folded proteins to the Golgi apparatus and retains unfolded or misfolded proteins [88]. Consequently, ER plays a key role in the general cellular response to altered environmental conditions, such as nutrient overload or deprivation, abnormal increase in synthesis of secretory proteins, expression of mutant or misfolded proteins, and microbial infections [88, 89]. All of these “stressor signals” can lead to disruption of ER homeostasis and accumulation of unfolded proteins in the lumen, a condition called ER stress. In order to adapt ER function to this stress, a highly conserved signaling pathway called the unfolded protein response (UPR), or the ER stress response, is activated [87, 89–91]. The activated UPR reduces the translocation of new proteins into the ER lumen and increases retrotranslocation and degradation of misfolded proteins, recovering the folding capacity of the ER. This integrated ER stress response is characterized by transcriptional activation of multiple UPR-responsive genes mediated by inositol-requiring enzyme 1 alpha (IRE1*α*) and activating transcription factor 6 (ATF6), promoting a general decrease in translation initiation and a selective translation of several specific mRNAs mediated by PKR-like ER-associated kinase (PERK) [87]. IRE1*α*, PERK, and ATF6, transmembrane proteins localized on ER surface, are referred to as UPR sensors. These proteins are normally bound by the ER chaperone immunoglobulin binding protein BiP/GRP-78 at intraluminal domains. When immature proteins (also bound by BiP) exceed ER folding capacity, less BiP is available for binding to the UPR sensors. As a consequence, without BiP binding, PERK and IRE-1*α* autooligomerize and undergo autophosphorylation, leading to the activation of downstream signaling. A key mediator of the UPR is the mRNA encoding to X-box DNA binding protein 1 (XBP1), which is cleaved by the cytosolic endoribonuclease motif of activated IRE-1*α*, allowing for translation of its mRNA and consequently the generation of XBP1, a potent transcription factor. Moreover, activated PERK leads to an attenuation of general protein synthesis through inhibitory serine phosphorylation of eukaryotic translational initiation factor 2*α* (eIF2*α*). Interestingly, serine phosphorylation of eIF2*α* also results in specific translation of ATF4, another nuclear UPR mediator. Moreover, the release of ATF-6 from BiP binding frees this UPR sensor to be translocated to the Golgi, where it completes its activation as a functional transcription factor. All of these transcription factors (XBP1, ATF4, and ATF6) are translocated to the nucleus where they are able to stimulate the expression of multiple genes implicated in the final adaptive effects of UPR. In this context, it has been reported that transcriptional stimulation of adaptive genes depends on availability of specific ER stress response elements (ERSE), unfolded protein response elements (UPRE), or amino acid response elements (AARE) in the promoter region. Under normal conditions, the UPR pathway functions as a physiological adaptive mechanism. In contrast, when the primary stimulus is too persistent or severe, the ER stress response can lead to irreversible cell damage and programmed death through stimulation of proapoptotic transcription factor growth arrest and DNA damage-inducible gene 153 (GADD153, also called C/EBP homologous protein or CHOP) [87, 89–91].

The UPR is considered an efficient cellular mechanism of adaptation to multiple physiological conditions, but it has also been implicated in the physiopathology of various diseases [86, 88–90]. Despite the fact that first descriptions of UPR elements (such as BiP and IRE1) were associated with genes upregulated by glucose starvation, the ER stress response pathway is also evoked by the nutrient overload observed in diabetes mellitus and obesity. Currently, it is widely accepted that UPR plays a key role in the pathogenesis of diabetes due to its participation in pancreatic *β*-cell loss and peripheral IR [17, 18, 20, 92]. Moreover, the UPR stimulates the transcription of glucose-regulated proteins that may provide a protective function by increasing cellular capacity related to uptake and use of glucose. Nonetheless, during chronic hyperglycemia or nutrient excess, *β*-cells are exposed to high levels of immature insulin accumulated in the ER lumen, which may induce cell death through UPR-related mechanisms [17]. Hence, the ER stress response would play a dual role, acting as a beneficial regulator under physiological conditions or triggering *β*-cell dysfunction and apoptosis under a chronic stress environment.

Interestingly, it has been reported that HFD and obesity induce ER stress in the liver, which suppresses insulin signaling via JNK activation, establishing a potential link between obesity and IR [15, 88]. Moreover, liver cells exposed to pharmacological triggers of ER stress response show IR profiles characterized by serine phosphorylation of IRS-1 and suppression of insulin-induced Akt phosphorylation. Since these alterations in the insulin pathway are blocked by inhibition of JNK, ER stress may promote a JNK-dependent serine phosphorylation of IRS-1, which in turn inhibits insulin receptor signaling. Further experiments confirm crucial roles for IRE1 as a promoter and XBP1 as an inhibitor of ER stress-associated insulin resistance in obesity [15, 93]. In addition, it has been reported that preventing ER stress in obese and diabetic mice with chemical chaperones (such as 4-phenyl butyric acid and taurine-conjugated ursodeoxycholic acid, TUDCA) was associated with restoration of insulin sensitivity at systemic and tissue levels (liver, muscle, and fat) [15, 94]. All of these results suggest that treating individuals exposed to an obesity-related condition with ER stress-alleviating compounds could be used as a new therapeutic tool to prevent or reverse the deleterious effects of obesity, insulin resistance, and pro-inflammatory markers.

## 5. Link between Inflammation and ER Stress-Related Insulin Resistance

The classic function of the immune system is defense against infections by detecting pathogen-associated molecular patterns (PAMPs), such as bacterial and viral components. However, immune cells are also able to sense damage associated with damage-associated molecular patterns (DAMPs), such as extracellular nucleotides and cytoplasmic and nuclear components [95]. After activation, immune cells use different mechanisms for cell-to-cell communication, including cytokines, which are mainly soluble proteins that can promote pro- or anti-inflammatory responses [96]. Cytokines are produced not only by immune cells but also by almost all cells and activate immune response during injury or infection. Abnormal release of cytokines can promote development and progression of various pathological conditions with diverse etiologies [96]. Moreover, obese individuals exhibit high levels of several pro-inflammatory cytokines, which promote an inflammatory state related to tissue damage [97, 98]. Currently, there is a rising interest regarding the role of inflammation during obesity, especially in cases where exercise and dietary treatment are insufficient to restore the nutritional state [99]. This situation is likely due to a chronic pro-inflammatory response, mediated by various pro-inflammatory cytokines that promote modulation of T cell function toward the Th1 phenotype and macrophage differentiation toward a deleterious M1 phenotype. In contrast to this effect, the predominance of anti-inflammatory cytokines in healthy nonobese individuals shifts T cell and macrophage polarization toward Th2 and M2 phenotypes, respectively [100]. After TNF-*α* was described as a major pro-inflammatory cytokine expressed in adipose tissue and with a relationship to IR in murine models of obesity [101], a rising number of studies showed that the immune system contributes to the sensing of metabolites and nutritional status in the whole body [84].

Inflammation has been related to ER stress development; nevertheless, controversy remains as to whether this cellular stress response promotes or prevents progression of several diseases [102]. Under obesity conditions, ER stress may have a deleterious effect associated with the pro-inflammatory state and induction of IR [20]. Interestingly, other pro-inflammatory cytokines directly affect both function and viability of *β*-pancreatic insulin-producing cells [103]. The adverse effects of TNF-*α*, IL-1*β*, and interferon *γ* (IFN-*γ*) are prevented when *β*-cells are treated with anti-inflammatory cytokines (IL-4, IL-10, and IL-13), showing that these molecules may modulate insulin serum levels, which in turn affect metabolic control at different levels. In addition, pro-inflammatory cytokines induce upregulation of ATF4 mRNA in *β*-pancreatic cells by disrupting Ca^2+^ signaling [104, 105]. Thus, because ATF4 is a classical effector of the PERK signaling cascade, a direct link between ER stress and inflammation has been proposed.

Indeed, ER stress is linked to cytokines because activation of ATF6 and cAMP-responsive element-binding protein hepatocyte specific (CREBH) is the main factor responsible for release of TNF-*α*, IL-1*β*, and IL-6 [102]. Adipose tissues from murine obesity models show increased mRNA levels of pro-inflammatory cytokines, which are restored to normal levels after treatment with TUDCA, a chemical chaperone that inhibits ER stress [106]. In addition, activation of ER stress-related protein PKR has been described in cells exposed to TNF-*α* [107], showing a direct induction of ER stress through this cytokine. In the same way, it has been suggested that IFN-*γ* may directly induce ER stress, because IFN-*γ* also activates PKR [108]. Furthermore, interferon regulatory factor 7 (IRF-7) was found to be a positive regulator of weight gain in a murine model [109], suggesting an obesity-related negative feedback cycle, depending on the interferon pathway. Moreover, PKR is involved in secretion of IL-1*β* and IL-18 [110], although the latter cytokine seems to prevent obesity and IR in mice [111]. We hypothesize that the final effect on insulin signaling may depend on the interaction among different pro- or anti-inflammatory cytokines and ER stress proteins at the systemic or microenvironmental level. Surprisingly, recent evidence has shown that insulin may increase ER stress markers in adipose tissue [112]. Therefore, this interesting new evidence suggests that ER stress may occur after development of IR, reinforcing the hypothesis regarding an obesity-inflammation-ER stress vicious cycle.

Unlike pro-inflammatory cytokines, anti-inflammatory molecules have been linked with prevention of ER stress development. Indeed, the major anti-inflammatory cytokine IL-10 has been related to impaired ATF6 nuclear translocation induced by both TNF-*α* [113] and tunicamycin [114], suggesting that mechanisms involved in ER stress inhibition by IL-10 may be independent of stress response. Thus, whether other anti-inflammatory cytokines, such as IL-4 or IL-13, are able to inhibit or prevent ER stress should be addressed. For example, it has been reported that IL-6 also inhibits obesity-induced ER stress in the rat hypothalamus [115]. Similarly, other anti-inflammatory agents, such as omega-3 fatty acids, may also produce insulin sensitization and antidiabetic effects (such as restoration of Akt signaling) through G protein-coupled receptor 120 (GPR120) [116]. Accordingly, it is possible that omega-3 fatty acids or other fatty acids may also inhibit ER stress.

Unexpectedly, a pro-inflammatory cytokine named resistin showed chaperone activity and was able to inhibit ER stress. Interestingly, this study demonstrated that resistin was retained inside the cell to inhibit ER stress, suggesting that soluble and cellular resistin may have different effects and cellular targets [117]. Klotho protein also promotes differential cellular effects in terms of insulin function and inflammation depending on the circulating or intracellular fraction of this aging suppressor protein [118, 119]. Intravenous administration of the soluble extracellular domain of Klotho, which is also found in the blood, binds to its putative receptor and inhibits the insulin pathway [120]. Furthermore, intracellular but not the secreted form of Klotho protein has an anti-inflammatory effect over retinoic acid inducible gene I (RIG-I) signaling and inhibits IL-6 and IL-8 release [121]. Recent evidence has also shown that overexpression of Klotho is able to inhibit chemically-induced ER stress [122]. The hypothesis of differential action depending on target location opens a new field for the study of cytokines, showing that soluble or intracellular cytokines may differentially modulate cellular responses in both physiological and pathophysiological conditions.

In the context of pregnancy, cytokines may significantly affect the metabolic state, which in turn promotes IR and a pro-inflammation condition associated with MO. Importantly, cytokine-induced fetal programming has been proposed in rats after maternal exposure to both TNF-*α* and IL-6 treatment, associated with increased fetal growth and IR in the offspring [123]. Moreover, IL-6 seems to play a pivotal role in the transference of a pro-inflammatory state from the mother to the fetus, as umbilical cord blood levels of IL-6 from obese mothers are higher than those from normal pregnancies [40]. This finding is also related to increased macrophage infiltration of placental tissue, associated with elevated pro-inflammatory markers in response to MO [124]. Interestingly, while pro-inflammatory cytokines can induce ER stress in placental tissue, an anti-inflammatory response may restore normal insulin sensitivity. Accordingly, administering an anti-inflammatory flavonoid named quercetin [125] during pregnancy and lactation significantly decreases ER stress activation in the offspring [126], suggesting that it may be possible to modulate the prenatal environment, preventing ER stress and its deleterious consequences.

Additional mechanisms related to cellular stress and/or inflammatory responses (such as maternal psychological environment, dietary behavior, and infections) could affect intrauterine development, highlighting the role of new players in obesity and immune system abnormalities associated with deleterious metabolic outcomes, both at the maternal and at the fetal levels. For example, PKR, which is an ER stress-dependent protein kinase, is also activated by viral infections and is characterized by inflammatory, IR, and ER stress responses [107]. In fact, inhibition of PKR has been associated with decreased expression of ER stress markers and improved insulin sensitivity in obese/diabetic mice, involving reduction of inflammation [127]. This finding suggests that PKR may play a key role as a pharmacological target in metabolic diseases under obesity conditions. Mental stress during pregnancy should also be considered as an initial risk factor related to obesity and IR development [128]. Neuroendocrine interactions with important roles in depression and sickness [129] are associated with impaired anorexigenic signaling and obesity tendencies in fetuses from mothers with MO [130]. Thus, it may be relevant to also consider inflammation-related processes, such as infection or mental stress during pregnancy, as potential risk factors contributing to fetal programming of metabolic diseases.

**Figure 1:**
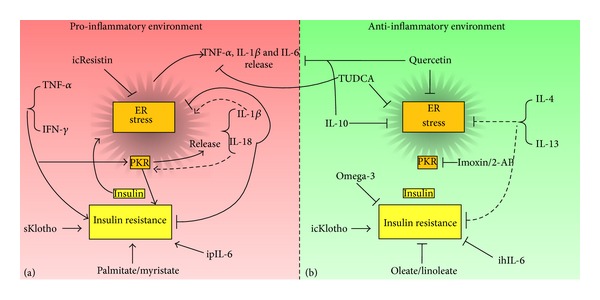
Balance of pro-inflammatory and anti-inflammatory cytokines in the development of ER stress and insulin resistance. In this scheme we highlight the role of several pro-inflammatory cytokines (a) and anti-inflammatory cytokines (b). These models integrate information from animal and cellular different models, which can be extrapolated to other systems. Cytokines or other molecules with pro-inflammatory effects have a tendency to induce the ER stress and produce insulin resistance. Interestingly, cytokines or other molecules with anti-inflammatory effects, such as TUDCA, omega 3 fatty acids, or quercetin, prevent the release of pro-inflammatory cytokines, inhibit the development of ER stress, and induce insulin sensitizing, improving glucose metabolism (icResistin = intracellular resistin; sKlotho = soluble Klotho; icKlotho = intracellular Klotho; ipIL-6 = intraperitoneally injected IL-6; ihIL-6 = intrahypothalamically injected IL-6).

The balance between pro- and anti-inflammatory immune cell phenotypes may be modulated to avoid the deleterious immune imbalance that provokes metabolic alterations in pregnancies complicated by obesity. The complex interactions among multiple inflammatory mediators and the ER stress response should be considered in the study of fetal IR development attributable to MO (Figure 1).

## 6. Fetal Programming of Insulin Resistance by Maternal Obesity-Dependent ER Stress

**Figure 2:**
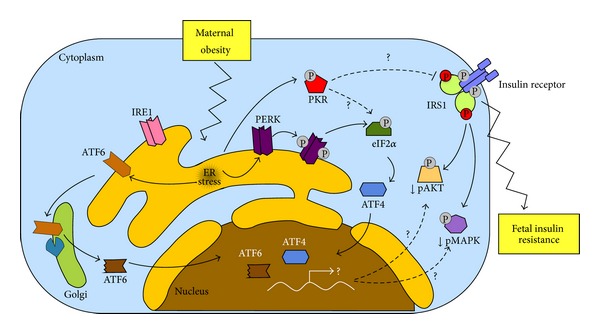
Proposed model of interaction among maternal obesity, ER stress, and insulin resistance. Maternal obesity is related to ER stress response in HUVEC, involving activation of ER stress proteins PERK and ATF6. ATF6 is released from ER membranes and then processed in the Golgi by proteolytic cleavage promoting its nuclear translocation. On the other hand, PERK is autophosphorylated (grey circles) and is able to phosphorylate eIF2*α*, leading to induction of ATF4. Moreover, eIF2*α* can also be phosphorylated by PKR, which is also an ER stress-dependent protein. Hence, both ATF6 and ATF4 nuclear translocations may be able to alter insulin signaling and lead to insulin resistance in HUVEC through reduction of AKT and MAPK phosphorylation. In parallel, PKR activation may cause insulin signaling inactivation through IRS-1 inhibitory phosphorylation (red circles). Solid lines represent previously established processes; dashed lines and question marks indicate hypothetical and unknown mechanisms in our model.

As compared to normal pregnancy, MO is associated with an exaggerated lipid mobilization (increased plasma cholesterol and triacylglycerol) and abnormal accumulation of fat in the liver, pancreas, and placenta [40, 131]. In addition, obesity in pregnancy is related to increased IR [132], higher levels of inflammatory markers, and impaired endothelial function [71, 124, 133]. Moreover, maternal metabolic abnormalities associated with overnutrition during pregnancy may be transmitted to the fetal circulation, since fetal offspring from HFD-fed pregnant nonhuman primates showed increased markers of metabolic disorders associated with obesity, such as hepatic oxidative stress and nonalcoholic fatty liver disease (NAFLD) [134]. In this study, offspring of HFD pregnant animals also exhibited elevated hepatic expression of gluconeogenic enzymes and transcription factors, in addition to increased levels of plasma glycerol and liver triglycerides [134]. Consequently, these results suggest that stressor effects related to nutrient excess from maternal overfeeding are mimicked in fetal plasma and can produce fat-related liver disease in the offspring. Nevertheless, although ER stress has been implicated in conditions from hepatic steatosis to NAFLD [135], there is no evidence regarding the potential role of the endoplasmic reticulum in the pathological process described in the fetal liver from murine MO models. On the other hand, increasing epidemiological evidence has suggested intrauterine programming of IR in offspring from obese pregnant woman, evaluated both at an early neonatal stage and in young adulthood. Nevertheless, the mechanistic link between MO and offspring IR remains unclear. Since IR has been described as a keystone in physiopathology pathways associated with metabolic diseases such as diabetes and cardiovascular complications, the potential therapeutic target of ER stress during the early neonatal period or during pregnancy may be relevant to obstetric and postnatal outcomes. Accordingly, potential crosstalk between insulin signaling and ER stress pathways on human fetal cells exposed to maternal obesity is proposed (Figure 2).

Regarding the nutritional programming hypothesis, significant data have shown increased cardiometabolic risk in offspring from both under- and overnutrition in pregnancy. In overfed pregnant mouse models, fetal liver shows excessive lipid and fatty acid accumulation associated with activation of JNK, an oxidative stress, inflammatory, and apoptosis marker [134]. Hence, JNK activation and apoptosis are described as part of the ER stress pathway related to both IR and diabetes in response to obesity in various models. Therefore, it is possible that future interventions focused on preventing obesity-derived ER stress in pregnancy may target avoidance of IR development in fetal tissues. Although McCurdy et al. showed that prepregnancy diet normalization partially attenuated development of fatty liver disease in fetal offspring, there is no evidence regarding the potential beneficial effect of this nutritional intervention on fetal UPR or insulin sensitivity. Specific therapeutic interventions with chemical chaperones such as bile acids have shown improved hepatic insulin response in obese individuals [136]. However, although some bile acids are currently used in cases of icteric cholestasis of pregnancy [137], it remains unclear whether this treatment will be useful in preventing insulin resistance in the offspring of pregnancies with MO.

## 7. Conclusions

MO and neonatal IR are associated with long-term development of obesity, diabetes mellitus, and increased global cardiovascular risk in the offspring, involving deleterious mechanisms of intrauterine programming. Nevertheless, the entire signaling link among these conditions has not been fully elucidated. Recent evidence suggests that obesity-related ER stress may play an important role in the development of IR, associated with unfolded protein response (UPR) and inflammatory mediators. We propose a potential mechanism of MO-dependent ER stress response on human fetal cells, involving inflammatory cytokines such as TNF-*α*, IL-1*β*, IL-6, and/or IFN-*γ*, and activation of PERK, eIF2*α*, PKR, ATF4, and ATF6. Understanding this phenomenon may provide crucial information that would clarify the potential beneficial effects of new therapeutic tools to prevent the deleterious consequences associated with MO, inflammatory markers, and IR in the offspring.

## Figures and Tables

**Table 1:** Cardiovascular risk factors in offspring of pregnancies with maternal obesity.

Parameters	Effect	Maternal obesity	Offspring age (years)	Reference
Blood pressure	Increased	eGWG	9, 21	[33, 34]
		ePPW	Neonates, 6, 17	[35–38]
Body fat	Increased	eGWG	9	[33]
		ePPW	Neonates, 6	[37–40]
BMI	Increased	eGWG	9, 21	[33, 34]
		ePPW	6, 17	[36, 37]
IL-6	Increased	eGWG	9	[33]
		ePPW	Neonates	[40]
CRP	Increased	eGWG	9	[33]
Abdominal fat	Increased	eGWG	9	[33]
		ePPW	6	[37]
Leptin	Increased	eGWG	9	[33]
		ePPW	Neonates	[40]
HDL	Decreased	eGWG	9	[33]
		ePPW	6	[37]
ApoA1	Decreased	eGWG	9	[33]
Insulin	Increased	ePPW	6	[37]
HOMA-IR	Increased	ePPW	Neonates	[40]

ePPW, excessive prepregnancy weight; eGWG, excessive gestational weight gain; BMI, body mass index; IL-6, interleukin 6; CRP; c-reactive protein; HDL: high-density lipoprotein; ApoA1: apolipoprotein A-I; HOMA-IR: homeostasis model assessment for insulin resistance.

## References

[1] WHO (2003). Diet, nutrition and the prevention of chronic diseases. *WHO Technical Report*.

[2] WHO (2011). *WHO Global Database on Body Mass Index*.

[3] Triunfo S, Lanzone A (2014). Impact of overweight and obesity on obstetric outcomes. *Journal of Endocrinological Investigation*.

[4] ACOG (2005). ACOG Committee Opinion number 315, September 2005. Obesity in pregnancy. *Obstetrics and Gynecology*.

[5] Flenady V, Koopmans L, Middleton P (2011). Major risk factors for stillbirth in high-income countries: a systematic review and meta-analysis. *The Lancet*.

[6] Taylor PD, Samuelsson AM, Poston L (2014). Maternal obesity and the developmental programming of hypertension: a role for leptin. *Acta Physiologica*.

[7] Dong M, Zheng Q, Ford SP, Nathanielsz PW, Ren J (2013). Maternal obesity, lipotoxicity and cardiovascular diseases in offspring. *Journal of Molecular and Cellular Cardiology*.

[8] Deng T, Lyon CJ, Minze LJ (2013). Class II major histocompatibility complex plays an essential role in obesity-induced adipose inflammation. *Cell Metabolism*.

[9] Han MS, Jung DY, Morel C (2013). JNK expression by macrophages promotes obesity-induced insulin resistance and inflammation. *Science*.

[10] Matsuda M, Shimomura I (2013). Increased oxidative stress in obesity: implications for metabolic syndrome, diabetes, hypertension, dyslipidemia, atherosclerosis, and cancer. *Obesity Research and Clinical Practice*.

[11] Cawthorn WP, Sethi JK (2008). TNF-*α* and adipocyte biology. *FEBS Letters*.

[12] Ouchi N, Parker JL, Lugus JJ, Walsh K (2011). Adipokines in inflammation and metabolic disease. *Nature Reviews Immunology*.

[13] Cao H (2014). Adipocytokines in obesity and metabolic disease. *Journal of Endocrinology*.

[14] Flamment M, Hajduch E, Ferré P, Foufelle F (2012). New insights into ER stress-induced insulin resistance. *Trends in Endocrinology and Metabolism*.

[15] Özcan U, Cao Q, Yilmaz E (2004). Endoplasmic reticulum stress links obesity, insulin action, and type 2 diabetes. *Science*.

[16] Hansen PA, Waheed A, Corbett JA (2007). Chemically chaperoning the actions of insulin. *Trends in Endocrinology and Metabolism*.

[17] Eizirik DL, Cardozo AK, Cnop M (2008). The role for endoplasmic reticulum stress in diabetes mellitus. *Endocrine Reviews*.

[18] Schenk S, Saberi M, Olefsky JM (2008). Insulin sensitivity: Modulation by nutrients and inflammation. *Journal of Clinical Investigation*.

[19] Zeyda M, Stulnig TM (2009). Obesity, inflammation, and insulin resistance—a mini-review. *Gerontology*.

[20] Hotamisligil GS (2010). Endoplasmic Reticulum Stress and the Inflammatory Basis of Metabolic Disease. *Cell*.

[21] Yalcin A, Hotamisligil GS (2013). Impact of ER protein homeostasis on metabolism. *Diabetes*.

[22] Lee J, Ozcan U (2014). Unfolded protein response signaling and metabolic diseases. *The Journal of Biological Chemistry*.

[23] Tenenbaum-Gavish K, Hod M (2013). Impact of maternal obesity on fetal health. *Fetal Diagnosis and Therapy*.

[24] IOM (1990). *Nutrition during Pregnancy*.

[25] Papachatzi E, Dimitriou G, Dimitropoulos K, Vantarakis A (2013). Pre-pregnancy obesity: maternal, neonatal and childhood outcomes. *Journal of Neonatal- Perinatal Medicine*.

[26] Catalano PM (2003). Obesity and pregnancy—the propagation of a viscous cycle?. *The Journal of Clinical Endocrinology & Metabolism*.

[27] Catalano PM, Mele L, Landon MB (2014). Inadequate weight gain in overweight and obese pregnant women: what is the effect on fetal growth?. *American Journal of Obstetrics and Gynecology*.

[28] Curhan GC, Willett WC, Rimm EB, Spiegelman D, Ascherio AL, Stampfer MJ (1996). Birth weight and adult hypertension, diabetes mellitus, and obesity in US men. *Circulation*.

[29] Öken E, Rifas-Shiman SL, Field AE, Frazier AL, Gillman MW (2008). Maternal gestational weight gain and offspring weight in adolescence. *Obstetrics and Gynecology*.

[30] Birbilis M, Moschonis G, Mougios V, Manios Y (2013). Obesity in adolescence is associated with perinatal risk factors, parental BMI and sociodemographic characteristics. *European Journal of Clinical Nutrition*.

[31] Oken E, Taveras EM, Kleinman KP, Rich-Edwards JW, Gillman MW (2007). Gestational weight gain and child adiposity at age 3 years. *The American Journal of Obstetrics and Gynecology*.

[32] Wrotniak BH, Shults J, Butts S, Stettler N (2008). Gestational weight gain and risk of overweight in the offspring at age 7 y in a multicenter, multiethnic cohort study. *The American Journal of Clinical Nutrition*.

[33] Fraser A, Tilling K, MacDonald-Wallis C (2010). Association of maternal weight gain in pregnancy with offspring obesity and metabolic and vascular traits in childhood. *Circulation*.

[34] Mamun AA, O'Callaghan M, Callaway L, Williams G, Najman J, Lawlor DA (2009). Associations of gestational weight gain with offspring body mass index and blood pressure at 21 years of ageevidence from a birth cohort study. *Circulation*.

[35] Margetts BM, Rowland MGM, Foord FA, Cruddas AM, Cole TJ, Barker DJP (1991). The relation of maternal weight to the blood pressures of Gambian children. *International Journal of Epidemiology*.

[36] Laor A, Stevenson DK, Shemer J, Gale R, Seidman DS (1997). Size at birth, maternal nutritional status in pregnancy, and blood pressure at age 17: population based analysis. *British Medical Journal*.

[37] Gaillard R, Steegers EA, Duijts L (2014). Childhood cardiometabolic outcomes of maternal obesity during pregnancy: The Generation R Study. *Hypertension*.

[38] Stuebe AM, Landon MB, Lai Y (2012). Maternal BMI, glucose tolerance, and adverse pregnancy outcomes. *American Journal of Obstetrics & Gynecology*.

[39] Sewell MF, Huston-Presley L, Super DM, Catalano P (2006). Increased neonatal fat mass, not lean body mass, is associated with maternal obesity. *American Journal of Obstetrics and Gynecology*.

[40] Catalano PM, Presley L, Minium J, Hauguel-de Mouzon S (2009). Fetuses of obese mothers develop insulin resistance in utero. *Diabetes Care*.

[41] Öken E, Gillman MW (2003). Fetal origins of obesity. *Obesity Research*.

[42] Ehrenberg HM, Mercer BM, Catalano PM (2004). The influence of obesity and diabetes on the prevalence of macrosomia. *American Journal of Obstetrics & Gynecology*.

[43] Dörner G, Plagemann A (1994). Perinatal hyperinsulinism as possible predisposing factor for diabetes mellitus, obesity and enhanced cardiovascular risk in later life. *Hormone and Metabolic Research*.

[44] O'Reilly JR, Reynolds RM (2013). The risk of maternal obesity to the long-term health of the offspring. *Clinical Endocrinology*.

[45] Godfrey KM, Forrester T, Barker DJP (1994). Maternal nutritional status in pregnancy and blood pressure in childhood. *British Journal of Obstetrics and Gynaecology*.

[46] Clark PM, Atton C, Law CM, Shiell A, Godfrey K, Barker DJP (1998). Weight gain in pregnancy, triceps skinfold thickness, and blood pressure, in offspring. *Obstetrics and Gynecology*.

[47] Ryckman KK, Borowski KS, Parikh NI, Saftlas AF (2013). Pregnancy complications and the risk of metabolic syndrome for the offspring. *Current Cardiovascular Risk Reports*.

[48] Magarey AM, Daniels LA, Boulton TJ, Cockington RA (2003). Predicting obesity in early adulthood from childhood and parental obesity. *International Journal of Obesity and Related Metabolic Disorders*.

[49] Danielzik S, Czerwinski-Mast M, Langnäse K, Dilba B, Müller MJ (2004). Parental overweight, socioeconomic status and high birth weight are the major determinants of overweight and obesity in 5-7 y-old children: baseline data of the Kiel Obesity Prevention Study (KOPS). *International Journal of Obesity*.

[50] Salsberry PJ, Reagan PB (2005). Dynamics of early childhood overweight. *Pediatrics*.

[51] Adamo KB, Ferraro ZM, Goldfield G (2013). The Maternal Obesity Management (MOM) Trial Protocol: a lifestyle intervention during pregnancy to minimize downstream obesity. *Contemporary Clinical Trials*.

[52] Ainge H, Thompson C, Ozanne SE, Rooney KB (2011). A systematic review on animal models of maternal high fat feeding and offspring glycaemic control. *International Journal of Obesity*.

[53] Li M, Sloboda DM, Vickers MH (2011). Maternal obesity and developmental programming of metabolic disorders in offspring: Evidence from animal models. *Experimental Diabetes Research*.

[54] Guo F, Jen K-LC (1995). High-fat feeding during pregnancy and lactation affects offspring metabolism in rats. *Physiology and Behavior*.

[55] Samuelsson A, Matthews PA, Argenton M (2008). Diet-induced obesity in female mice leads to offspring hyperphagia, adiposity, hypertension, and insulin resistance: a novel murine model of developmental programming. *Hypertension*.

[56] Oben JA, Mouralidarane A, Samuelsson AM (2010). Maternal obesity during pregnancy and lactation programs the development of offspring non-alcoholic fatty liver disease in mice. *Journal of Hepatology*.

[57] Han J, Xu J, Epstein PN, Liu YQ (2005). Long-term effect of maternal obesity on pancreatic beta cells of offspring: reduced beta cell adaptation to high glucose and high-fat diet challenges in adult female mouse offspring. *Diabetologia*.

[58] Yamashita H, Shao J, Qiao L, Pagliassotti M, Friedman JE (2003). Effect of spontaneous gestational diabetes on fetal and postnatal hepatic insulin resistance in Leprdb/+ mice. *Pediatric Research*.

[59] Metzger BE, Silverman BL, Freinkel N, Dooley SL, Ogata ES, Green OC (1990). Amniotic fluid insulin concentration as a predictor of obesity. *Archives of Disease in Childhood*.

[60] Boskovic R, Feig DS, Derewlany L, Knie B, Portnoi G, Koren G (2003). Transfer of insulin lispro across the human placenta: in vitro perfusion studies. *Diabetes Care*.

[61] Holcberg G, Tsadkin-Tamir M, Sapir O (2004). Transfer of insulin lispro across the human placenta. *European Journal of Obstetrics Gynecology and Reproductive Biology*.

[62] Burton GJ, Fowden AL (2012). Review: the placenta and developmental programming: Balancing fetal nutrient demands with maternal resource allocation. *Placenta*.

[63] Pedersen J (1977). *The Pregnant Diabetic and Her Newborn*.

[64] Westermeier F, Salomón C, González M (2011). Insulin restores gestational diabetes mellitus-reduced adenosine transport involving differential expression of insulin receptor isoforms in human umbilical vein endothelium. *Diabetes*.

[65] Salomón C, Westermeier F, Puebla C (2012). Gestational diabetes reduces adenosine transport in human placental microvascular endothelium, an effect reversed by insulin. *PLoS ONE*.

[66] Leung TW, Lao TT (2000). Placental size and large-for-gestational-age infants in women with abnormal glucose tolerance in pregnancy. *Diabetic Medicine*.

[67] Jones AP, Pothos EN, Rada P, Olster DH, Hoebel BG (1995). Maternal hormonal manipulations in rats cause obesity and increase medial hypothalamic norepinephrine release in male offspring. *Developmental Brain Research*.

[68] Plagemann A, Heidrich I, Götz F, Rohde W, Dorner G (1992). Lifelong enhanced diabetes susceptibility and obesity after temporary intrahypothalamic hyperinsulinism during brain organization. *Experimental and Clinical Endocrinology*.

[69] Hauguel-de Mouzon S, Lepercq J, Catalano P (2006). The known and unknown of leptin in pregnancy. *American Journal of Obstetrics & Gynecology*.

[70] Smith JT, Waddell BJ (2003). Leptin distribution and metabolism in the pregnant rat: transplacental leptin passage increases in late gestation but is reduced by excess glucocorticoids. *Endocrinology*.

[71] Stewart FM, Freeman DJ, Ramsay JE, Greer IA, Caslake M, Ferrell WR (2007). Longitudinal assessment of maternal endothelial function and markers of inflammation and placental function throughout pregnancy in lean and obese mothers. *The Journal of Clinical Endocrinology and Metabolism*.

[72] Meshkani R, Adeli K (2009). Hepatic insulin resistance, metabolic syndrome and cardiovascular disease. *Clinical Biochemistry*.

[73] Muniyappa R, Montagnani M, Koh KK, Quon MJ (2007). Cardiovascular actions of insulin. *Endocrine Reviews*.

[74] Ullrich A, Bell JR, Chen EY (1985). Human insulin receptor and its relationship to the tyrosine kinase family of oncogenes. *Nature*.

[75] Ebina Y, Edery M, Ellis L (1985). Expression of a functional human insulin receptor from a cloned cDNA in Chinese hamster ovary cells. *Proceedings of the National Academy of Sciences of the United States of America*.

[76] Belfiore A, Frasca F, Pandini G, Sciacca L, Vigneri R (2009). Insulin receptor isoforms and insulin receptor/insulin-like growth factor receptor hybrids in physiology and disease. *Endocrine Reviews*.

[77] Saltiel AR, Kahn CR (2001). Insulin signalling and the regulation of glucose and lipid metabolism. *Nature*.

[78] Kim J-A, Montagnani M, Kwang KK, Quon MJ (2006). Reciprocal relationships between insulin resistance and endothelial dysfunction: molecular and pathophysiological mechanisms. *Circulation*.

[79] Taniguchi CM, Emanuelli B, Kahn CR (2006). Critical nodes in signalling pathways: insights into insulin action. *Nature Reviews. Molecular Cell Biology*.

[80] Desoye G, Hauguel-de Mouzon S (2007). The human placenta in gestational diabetes mellitus: the insulin and cytokine network. *Diabetes Care*.

[81] Goodpaster BH (2013). Mitochondrial deficiency is associated with insulin resistance. *Diabetes*.

[82] Eringa EC, Stehouwer CDA, van Nieuw Amerongen GP, Ouwehand L, Westerhof N, Sipkema P (2004). Vasoconstrictor effects of insulin in skeletal muscle arterioles are mediated by ERK1/2 activation in endothelium. *American Journal of Physiology—Heart and Circulatory Physiology*.

[83] Czaja MJ (2010). JNK regulation of hepatic manifestations of the metabolic syndrome. *Trends in Endocrinology and Metabolism*.

[84] Odegaard JI, Chawla A (2013). The immune system as a sensor of the metabolic state. *Immunity*.

[85] Aye ILMH, Powell TL, Jansson T (2013). Review: adiponectin—The missing link between maternal adiposity, placental transport and fetal growth?. *Placenta*.

[86] Hirosumi J, Tuncman G, Chang L (2002). A central, role for JNK in obesity and insulin resistance. *Nature*.

[87] Marciniak SJ, Ron D (2006). Endoplasmic reticulum stress signaling in disease. *Physiological Reviews*.

[88] Cnop M, Foufelle F, Velloso LA (2012). Endoplasmic reticulum stress, obesity and diabetes. *Trends in Molecular Medicine*.

[89] Ron D, Walter P (2007). Signal integration in the endoplasmic reticulum unfolded protein response. *Nature Reviews Molecular Cell Biology*.

[90] Zhang K, Kaufman RJ (2006). Protein folding in the endoplasmic reticulum and the unfolded protein response. *Handbook of Experimental Pharmacology*.

[91] Hetz C (2012). The unfolded protein response: controlling cell fate decisions under ER stress and beyond. *Nature Reviews Molecular Cell Biology*.

[92] Chan JY, Luzuriaga J, Bensellam M, Biden TJ, Laybutt DR (2013). Failure of the adaptive unfolded protein response in islets of obese mice is linked with abnormalities in *β*-cell gene expression and progression to diabetes. *Diabetes*.

[93] Zhou Y, Lee J, Reno CM (2011). Regulation of glucose homeostasis through a XBP-1-FoxO1 interaction. *Nature Medicine*.

[94] Cha BH, Kim JS, Ahn JC (2014). The role of tauroursodeoxycholic acid on adipogenesis of human adipose-derived stem cells by modulation of ER stress. *Biomaterials*.

[95] Kono H, Rock KL (2008). How dying cells alert the immune system to danger. *Nature Reviews Immunology*.

[96] Dinarello CA (2007). Historical insights into cytokines. *European Journal of Immunology*.

[97] Hotamisligil GS, Erbay E (2008). Nutrient sensing and inflammation in metabolic diseases. *Nature Reviews Immunology*.

[98] Lumeng CN, Saltiel AR (2011). Inflammatory links between obesity and metabolic disease. *The Journal of Clinical Investigation*.

[99] Snyder-Cappione JE, Nikolajczyk BS (2013). When diet and exercise are not enough, think immunomodulation. *Molecular Aspects of Medicine*.

[100] Hummasti S, Hotamisligil GS (2010). Endoplasmic reticulum stress and inflammation in obesity and diabetes. *Circulation Research*.

[101] Hotamisligil GS, Shargill NS, Spiegelman BM (1993). Adipose expression of tumor necrosis factor-*α*: direct role in obesity-linked insulin resistance. *Science*.

[102] Garg AD, Kaczmarek A, Krysko O, Vandenabeele P, Krysko DV, Agostinis P (2012). ER stress-induced inflammation: does it aid or impede disease progression?. *Trends in Molecular Medicine*.

[103] Souza KLA, Gurgul-Convey E, Elsner M, Lenzen S (2008). Interaction between pro-inflammatory and anti-inflammatory cytokines in insulin-producing cells. *The Journal of Endocrinology*.

[104] Cardozo AK, Ortis F, Storling J (2005). Cytokines downregulate the sarcoendoplasmic reticulum pump Ca^2+^ ATPase 2b and deplete endoplasmic reticulum Ca^2+^, leading to induction of endoplasmic reticulum stress in pancreatic *β*-cells. *Diabetes*.

[105] O'Neill CM, Lu C, Corbin KL (2013). Circulating levels of IL-1B+IL-6 cause ER stress and dysfunction in islets from prediabetic male mice. *Endocrinology*.

[106] Kawasaki N, Asada R, Saito A, Kanemoto S, Imaizumi K (2012). Obesity-induced endoplasmic reticulum stress causes chronic inflammation in adipose tissue. *Scientific Reports*.

[107] Nakamura T, Furuhashi M, Li P (2010). Double-stranded RNA-dependent protein kinase links pathogen sensing with stress and metabolic homeostasis. *Cell*.

[108] Sharma B, Altman JK, Goussetis DJ, Verma AK, Platanias LC (2011). Protein kinase R as mediator of the effects of interferon (IFN) *γ* and tumor necrosis factor (TNF) *α* on normal and dysplastic hematopoiesis. *The Journal of Biological Chemistry*.

[109] Wang X, Zhang R, Zhang S (2013). Interferon regulatory factor 7 deficiency prevents diet-induced obesity and insulin resistance. *American Journal of Physiology—Endocrinology and Metabolism*.

[110] Lu B, Nakamura T, Inouye K (2012). Novel role of PKR in inflammasome activation and HMGB1 release. *Nature*.

[111] Netea MG, Joosten LA, Lewis E (2006). Deficiency of interleukin-18 in mice leads to hyperphagia, obesity and insulin resistance. *Nature Medicine*.

[112] Boden G, Cheung P, Salehi S (2014). Insulin regulates the unfolded protein response in human adipose tissue. *Diabetes*.

[113] Shkoda A, Ruiz PA, Daniel H (2007). Interleukin-10 blocked endoplasmic reticulum stress in intestinal epithelial cells: impact on chronic inflammation. *Gastroenterology*.

[114] Hasnain SZ, Tauro S, Das I (2013). IL-10 promotes production of intestinal mucus by suppressing protein misfolding and endoplasmic reticulum stress in goblet cells. *Gastroenterology*.

[115] Ropelle ER, Flores MB, Cintra DE (2010). IL-6 and IL-10 anti-inflammatory activity links exercise to hypothalamic insulin and leptin sensitivity through IKK*β* and ER stress inhibition. *PLoS Biology*.

[116] Oh DY, Talukdar S, Bae EJ (2010). GPR120 is an omega-3 fatty acid receptor mediating potent anti-inflammatory and insulin-sensitizing effects. *Cell*.

[117] Suragani M, Aadinarayana VD, Pinjari AB (2013). Human resistin, a proinflammatory cytokine, shows chaperone-like activity. *Proceedings of the National Academy of Sciences of the United States of America*.

[118] Kuro-o M, Matsumura Y, Aizawa H (1997). Mutation of the mouse klotho gene leads to a syndrome resembling ageing. *Nature*.

[119] Razzaque MS (2012). The role of Klotho in energy metabolism. *Nature Reviews Endocrinology*.

[120] Kurosu H, Yamamoto M, Clark JD (2005). Physiology: suppression of aging in mice by the hormone Klotho. *Science*.

[121] Liu F, Wu S, Ren H, Gu J (2011). Klotho suppresses RIG-I-mediated senescence-associated inflammation. *Nature Cell Biology*.

[122] Banerjee S, Zhao Y, Sarkar PS, Rosenblatt KP, Tilton RG, Choudhary S (2013). Klotho ameliorates chemically induced endoplasmic reticulum (ER) stress signaling. *Cellular Physiology and Biochemistry*.

[123] Dahlgren J, Nilsson C, Jennische E (2001). Prenatal cytokine exposure results in obesity and gender-specific programming. *American Journal of Physiology—Endocrinology and Metabolism*.

[124] Challier JC, Basu S, Bintein T (2008). Obesity in pregnancy stimulates macrophage accumulation and inflammation in the placenta. *Placenta*.

[125] Indra MR, Karyono S, Ratnawati R, Malik SG (2013). Quercetin suppresses inflammation by reducing ERK1/2 phosphorylation and NF kappa B activation in Leptin-induced human umbilical vein endothelial cells (HUVECs). *BMC Research Notes*.

[126] Wu Z, Zhao J, Xu H (2014). Maternal quercetin administration during gestation and lactation decrease endoplasmic reticulum stress and related inflammation in the adult offspring of obese female rats. *Europe Journal of Nutrition*.

[127] Nakamura T, Arduini A, Baccaro B, Furuhashi M, Hotamisligil GS (2014). Small-molecule inhibitors of PKR improve glucose homeostasis in obese diabetic mice. *Diabetes*.

[128] Entringer S, Buss C, Swanson JM (2012). Fetal programming of body composition, obesity, and metabolic function: the role of intrauterine stress and stress biology. *Journal of Nutrition and Metabolism*.

[129] Dantzer R, O’Connor JC, Freund GG, Johnson RW, Kelley KW (2008). From inflammation to sickness and depression: when the immune system subjugates the brain. *Nature Reviews Neuroscience*.

[130] Fisher RE, Steele M, Karrow NA (2012). Fetal programming of the neuroendocrine-immune system and metabolic disease. *Journal of Pregnancy*.

[131] Jarvie E, Hauguel-de-Mouzon S, Nelson SM, Sattar N, Catalano PM, Freeman DJ (2010). Lipotoxicity in obese pregnancy and its potential role in adverse pregnancy outcome and obesity in the offspring. *Clinical Science*.

[132] Catalano PM, Ehrenberg HM (2006). The short- and long-term implications of maternal obesity on the mother and her offspring. *British Journal of Obstetrics and Gynaecology*.

[133] Roberts KA, Riley SC, Reynolds RM (2011). Placental structure and inflammation in pregnancies associated with obesity. *Placenta*.

[134] McCurdy CE, Bishop JM, Williams SM (2009). Maternal high-fat diet triggers lipotoxicity in the fetal livers of nonhuman primates. *The Journal of Clinical Investigation*.

[135] Anderson N, Borlak J (2008). Molecular mechanisms and therapeutic targets in steatosis and steatohepatitis. *Pharmacological Reviews*.

[136] Kars M, Yang L, Gregor MF (2010). Tauroursodeoxycholic acid may improve liver and muscle but not adipose tissue insulin sensitivity in obese men and women. *Diabetes*.

[137] Roma MG, Toledo FD, Boaglio AC (2011). Ursodeoxycholic acid in cholestasis: linking action mechanisms to therapeutic applications. *Clinical Science*.

